# Collagen V Is a Potential Substrate for Clostridial Collagenase G in Pancreatic Islet Isolation

**DOI:** 10.1155/2016/4396756

**Published:** 2016-04-18

**Authors:** Hiroki Shima, Akiko Inagaki, Takehiro Imura, Youhei Yamagata, Kimiko Watanabe, Kazuhiko Igarashi, Masafumi Goto, Kazutaka Murayama

**Affiliations:** ^1^Department of Biochemistry, Tohoku University Graduate School of Medicine, Sendai 980-8575, Japan; ^2^AMED-CREST, Japan Agency for Medical Research and Development, Tokyo 100-0004, Japan; ^3^Division of Transplantation and Regenerative Medicine, Tohoku University School of Medicine, Sendai 980-8575, Japan; ^4^New Industry Creation Hatchery Center, Tohoku University, Sendai 980-8579, Japan; ^5^Graduate School of Agricultural Science, Tokyo University of Agriculture and Technology, Fuchu 183-8509, Japan; ^6^Center for Regulatory Epigenome and Diseases, Tohoku University Graduate School of Medicine, Sendai 980-8575, Japan; ^7^Graduate School of Biomedical Engineering, Tohoku University, Sendai 980-8575, Japan

## Abstract

The clostridial collagenases, H and G, play key roles in pancreatic islet isolation. Collagenases digest the peptide bond between Yaa and the subsequent Gly in Gly-Xaa-Yaa repeats. To fully understand the pancreatic islet isolation process, identification of the collagenase substrates in the tissue is very important. Although collagen types I and III were reported as possible substrates for collagenase H, the substrate for collagenase G remains unknown. In this study, collagen type V was focused upon as the target for collagenases.* In vitro* digestion experiments for collagen type V were performed and analyzed by SDS-PAGE and mass spectrometry. Porcine pancreatic tissues were digested* in vitro *under three conditions and observed during digestion. The results revealed that collagen type V was only digested by collagenase G and that the digestion was initiated from the N-terminal part. Tissue degradation during porcine islet isolation was only observed in the presence of both collagenases H and G. These findings suggest that collagen type V is one of the substrates for collagenase G. The enzymatic activity of collagenase G appears to be more important for pancreatic islet isolation in large mammals such as pigs and humans.

## 1. Introduction

**Figure 1:**
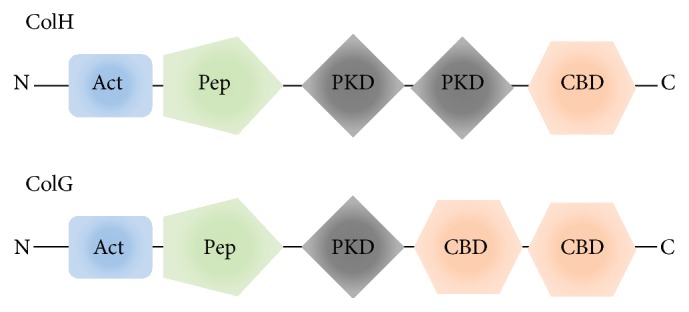
Schematic diagram of the domain architectures of ColG and ColH. Act: activator domain; Pep: peptidase domain; PKD: polycystic kidney disease-like domain; CBD: collagen-binding domain. The collagenase module is composed of two functional domains, an activator domain and a peptidase domain.

The clostridial collagenases, collagenase H (ColH) and collagenase G (ColG), are used for pancreatic islet isolation in combination with neutral proteases [1]. The collagenases possess a multimodular domain structure [2], and both collagenases have five domains (Figure 1). Activator and peptidase domains are involved in the collagenolytic activity [3] and are commonly contained in ColH and ColG. The active center comprises an HEXXH zinc-binding motif [4]. Polycystic kidney disease-like domains (PKDs) are located at the middle part of the enzyme, with two PKDs in ColH and one PKD in ColG. At the C-terminal end, both collagenases possess a collagenase binding domain (CBD), which is responsible for collagen triple helix binding [5, 6]. ColH has one CBD and ColG has two CBDs located tandemly. Molecular structures of clostridial collagenases have been revealed for the collagenase module of ColG [7], peptidase domain of ColH [8] (JBC, 288, 20184) and ColT [8], PKD of ColH [9] and ColG [7–10], and CBD of ColH [11] and ColG [11, 12]. Regarding the regulation of enzymatic activity, calcium ions play a critical role. The calcium ion-binding sites are contained within the PKD and CBD, and calcium ion binding enhances the stability of the protein molecule and maintains the domain conformations [13, 14]. The collagenases generally digest the bond between Yaa and Gly in the Gly-Xaa-Yaa(-Gly-) sequence repeats of fibrillar collagens [15, 16]. Degradation of collagen type I (Col-I) is well-known for collagenolytic activity of ColH and ColG. ColH also plays a crucial role during rat pancreatic islet isolation [17].* In vitro* pancreatic tissue digestion experiments and mass spectrometric analyses revealed that Col-I and collagen type III (Col-III) are substrates for ColH [17]. Although the coexistence of ColG can increase the yield of isolated islets, it has been difficult to determine the substrate specificity for ColG in rat experiments. Meanwhile, both ColH and ColG are used for pancreatic tissue dissociation in large mammals, such as pigs. Identification of the substrate for ColG in pancreatic tissue is one of the key factors toward understanding the pancreatic islet isolation process.

The fibrillar collagens are targets for many collagenases [2]. The commonly found fibrillar collagens are types I, II, III, and V [18]. A previous study showed that Col-I, collagen type II (Col-II), and Col-III were fully digested by ColG [17], while collagen type V (Col-V) was relatively less digested (manuscript in preparation). Col-V is a fibrillar collagen with low abundance. Several isoforms are found for Col-V, depending on the chain components [19]. The most common isoform is *α*1(V)_2_ *α*2(V). However, other isoforms such as *α*1(V)_3_ and *α*1(V)*α*2(V)*α*3(V) have also been reported [20]. Col-V exists in tissues colocalized with Col-I and appears to regulate the assembly of heterotypic fibers containing Col-I and Col-V [20–22].

In the present study, Col-V digestion by ColG was focused upon to investigate the substrate specificity of ColG. The digestions were analyzed by SDS-PAGE and mass spectrometry. The differences in Col-V digestion by ColH and ColG were evaluated. The results revealed that Col-V is a putative substrate for ColG in pancreatic islet isolation in large mammals.

## 2. Materials and Methods

### 2.1. Collagenases and Porcine Pancreatic Tissue

Recombinant enzymes of ColG and ColH, which were kindly provided by Meiji Seika Pharma Co. Ltd., were produced by an* Escherichia coli* protein expression system with ColG or ColH genes isolated from* Clostridium histolyticum*, as described previously [17].

Porcine pancreatic tissue samples were obtained from 2–4-year-old adult retired sows weighing 200–300 kg. All animal experiments were performed according to the guidelines for animal experiments and related activities at Tohoku University.

### 2.2. Digestion of Col-V

Col-V (from human placenta) was purchased from BD Biosciences (Bedford, MA). Enzyme solutions of ColH and ColG were prepared at 0.1 mg/mL with 100 mM HEPES (pH 8.0) and 1 mM CaCl_2_. Col-V (0.87 mg/mL) and enzyme solution (ColH or ColG) were mixed at a ratio of 1 : 1. The reactions were conducted at 30°C and sampled at 3, 6, 9, 12, 15, 30, 60, 120, and 240 min for sodium dodecyl sulfate-polyacrylamide gel electrophoresis (SDS-PAGE) and mass spectrometry. The sampling solutions were quenched by adding one-fifth volume of 50 mM ethylenediaminetetraacetic acid for SDS-PAGE and 1% trifluoroacetic acid for mass spectrometry. The SDS-PAGE gels were stained with Imperial Protein Stain (Thermo Fisher Scientific Inc., Waltham, MA). SDS-PAGE gel images for the lanes sampled at 60, 120, and 240 min were analyzed using Atto CS Analyzer 3 software (Atto Corporation, Tokyo, Japan).

The sampling solution at 60 min for ESI-MS was incubated at 60°C for 30 min and then digested (overnight) at 37°C with 10 *μ*g/mL trypsin adjusted to pH > 7 by sodium bicarbonate before digestion.

### 2.3. MALDI-tof-MS Analysis

MALDI-tof-MS analyses were carried out using an AXIMA performance mass spectrometer (Shimadzu, Kyoto, Japan) equipped with a 337 nm nitrogen laser operating in the positive-ion mode with an accelerating voltage of +20 kV. A timed ion selector was used to deflect ions of low* m/z* (<500) from the detector. The spectra were acquired by averaging the data from 200 laser shots to improve the data quality and ion statistics. The mass spectra were calibrated with angiotensin II and ACTH fragment 18–39 (Sigma) as external mass standards using *α*-cyano-4-hydroxycinnamic acid as a matrix. The spectra were processed using Biotech Launchpad software (Shimadzu).

### 2.4. Digestion of Porcine Pancreatic Tissue

Small pieces of porcine pancreatic tissue (~60 mg) were incubated with 0.1 mg/mL of thermolysin (Hampton Research, Aliso Viejo, CA) in 20 mM HEPES (pH 8.0) and 1 mM CaCl_2_ containing a protease inhibitor cocktail (complete; Roche, Basel, Switzerland) at 30°C overnight. The pancreatic tissues were then washed with the same buffer and further digested at 30°C with 0.1 mg/mL of ColH, ColG, or ColH + ColG for 9 h. The digestions were performed for three times for each condition. Each digested sample (100 *μ*L) was precipitated with ice-cold 10% trichloroacetic acid in acetone (400 *μ*L) and dried after centrifugation at 13,000 ×g. The dried samples were digested with trypsin (10 *μ*g/mL) at 37°C in 100 mM ammonium bicarbonate solution overnight.

### 2.5. Peptide Identification by ESI-MS

The digested peptides from the Col-V and tissue samples were treated with a ZipTip (Millipore, Billerica, MA) for mass spectrometry analysis and loaded onto a 75 *μ*m diameter 10 cm length fused-silica capillary column containing C18 resin (Nikkyo Technos, Tokyo, Japan). The peptides were eluted with an acetonitrile gradient (typically 2.4% to 22% in 46 min, then to 33% in 49 min) in 0.1% formic acid and analyzed by an LTQ Orbitrap Velos mass spectrometer (Thermo Fisher Scientific Inc.). Database searches against the NCBInr and Swissprot databases were performed using the MASCOT ver. 2.5.1 search engine (Matrix Science, London, UK). The MASCOT searches were carried out without protease specificity using 5 ppm peptide mass tolerance and 0.5 Da MS/MS tolerance. Oxidation (+15.9949 Da) was considered as a variable modification for Met, Cys, Pro, and Lys. The peptides identified with MASCOT expectation values < 0.05 were considered as significant hits.

## 3. Results

### 3.1. *In Vitro* Digestion of Col-V by Collagenases

#### 3.1.1. SDS-PAGE Analysis of Col-V Digestion

**Figure 2:**
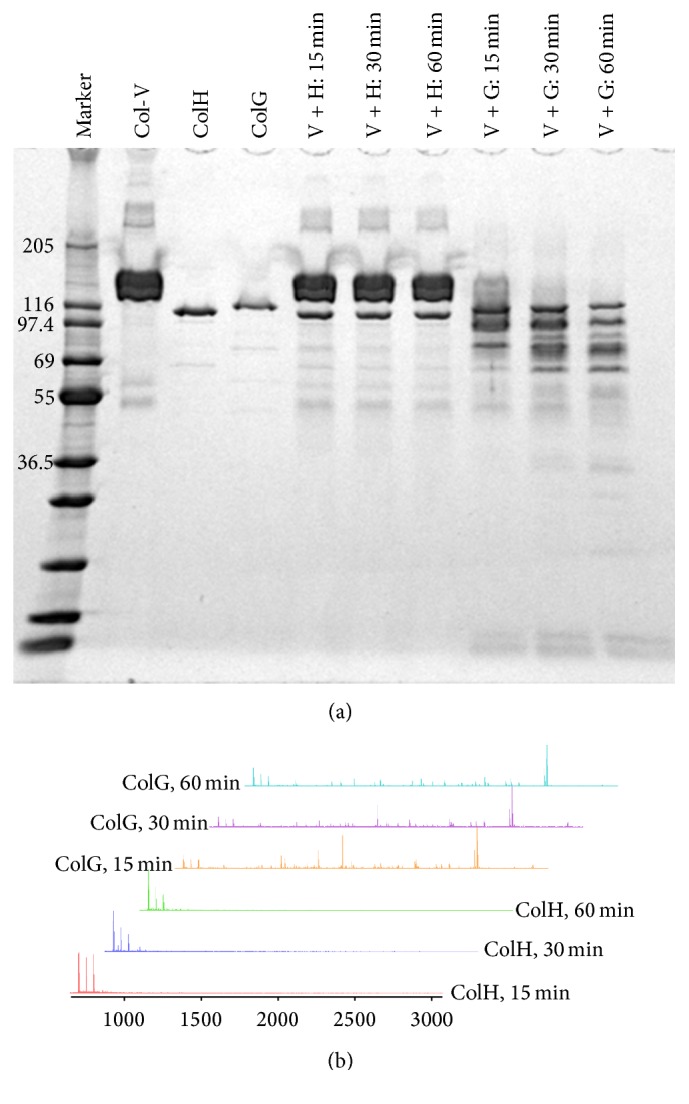
*In vitro* Col-V digestion by ColH and ColG. (a) SDS-PAGE analysis of digested Col-V. (b) MALDI-tof-MS analysis of the digested samples at 15, 30, and 60 min. The peak intensities are scaled to 100% for the most intense peak in each chart.

*In vitro* digestion of Col-V was performed to investigate the differences in digestion patterns by the collagenases.  Figure 2(a) shows an SDS-PAGE analysis of the Col-V digestion patterns by ColH and ColG from 15 to 60 min. The results showed distinct differences between the Col-V digestion patterns by ColH and ColG. On the SDS-PAGE gel, no band changes were observed in the lanes for ColH within 1 h, while ColG showed significant digestion of Col-V within this time frame.

#### 3.1.2. MALDI Analysis of Col-V Digestion

The SDS-PAGE analysis showed clear differences in the Col-V digestion patterns between ColH and ColG. Therefore, to sensitively detect the low molecular weight peptide fragments, MALDI-tof-MS analyses were conducted for the digestion solutions at 15, 30, and 60 min. The Col-V digestion samples by ColH indicated only a few peaks around* m/z* 700–800, which could reflect impurities arising from the matrix or originally present within the enzyme/Col-V solutions (Figure 2(b)). In contrast, the samples digested by ColG exhibited many peaks around* m/z* 1000–3000. These peaks were considered to be Col-V peptide fragments digested by ColG. Although the individual peak intensities could not be compared quantitatively in the MALDI spectra, the relative intensity patterns changed, indicating that the digestion sites by ColG changed over time.

### 3.2. Peptide Fragment Analysis by ESI-MS

To investigate the Col-V cleavage sites by ColG, the digested sample at 1 h was further fragmented by trypsin and analyzed by LC-MS. Peptide sequences and modifications (hydroxylated proline and lysine) were identified by MS/MS analysis. The MS experiments were performed in duplicate. The analyzed peptides included tryptic/semitryptic peptides (digested at the C-terminus of Lys/Arg), collagenolytic peptides (digested at the N-terminus of Gly-Xaa-Yaa repeats), and other peptides (neither tryptic nor collagenolytic peptides). The peptides were categorized tryptic peptides, semitryptic peptides including/not including Gly-Xaa-Yaa sequences at either side, and nontryptic peptides including/not including Gly-Xaa-Yaa sequences at either side or both sides. Considering this categorization, the total number of peptides was counted as the sum of the number of tryptic peptides + number of all semitryptic peptides + number of all nontryptic peptides. The collagenolytic peptides were calculated as the sum of the semitryptic peptides and nontryptic peptides including Gly-Xaa-Yaa sequences at either side or both sides. The results are summarized in Table 1. The total number of detected peptides was in the order of ColG/trypsin digestion > ColH/trypsin > trypsin. It was considered that the fragmentation of Col-V proceeded by double enzyme digestions, especially for the combination of ColG/trypsin. The Col-V digested by trypsin consisted of mainly tryptic and semitryptic peptides. Some tryptic peptides were shared with collagenolytic peptides, because the Col-V sequence included lysine or arginine at the Yaa position of the Gly-Xaa-Yaa repeats. The double-digested samples included both tryptic and collagenolytic peptides. In the ColH/trypsin digestion, the percentage of collagenolytic peptides was around 30% for *α*1 and 20% for *α*2. In contrast, the percentage of collagenolytic peptides for ColG/trypsin digestion exceeded 60–70% for all chain types. These results suggested that collagenolytic peptides were the main components in the ColG/trypsin digestion sample and therefore that ColG digested Col-V.

### 3.3. Initial Digestion of Col-V by ColG

#### 3.3.1. SDS-PAGE Analysis

**Figure 3:**
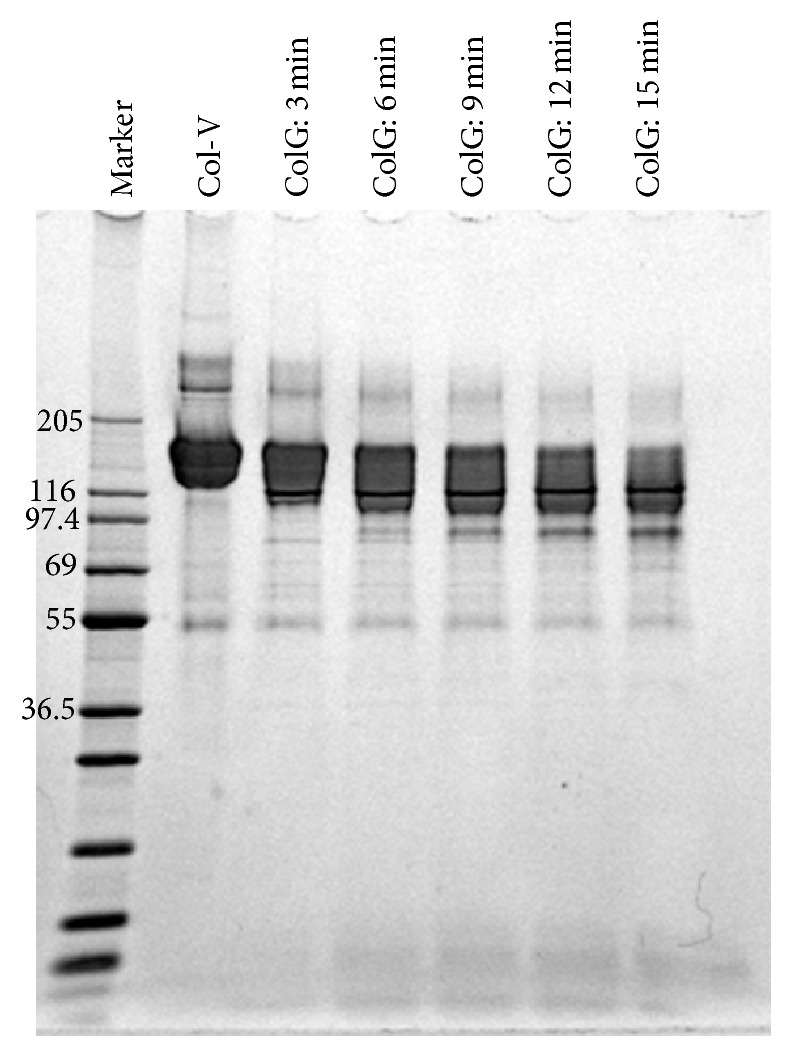
SDS-PAGE analysis of Col-V digestion by ColG within 15 min.

In this study,* in vitro* digestion was performed for 15–60 min, as described above. However, the Col-V digestion had already started by 15 min. To analyze the initial stage of the digestion, the Col-V digestion experiment was performed for a shorter time (3–15 min) and the digested samples were analyzed by SDS-PAGE (Figure 3). The main bands shifted to lower molecular weights over time. Furthermore, below the main bands, some new bands appeared, such as the band at ~95 kDa.

#### 3.3.2. Cleavage Site Distribution Analysis

The above results indicated that Col-V was degraded by ColG and therefore that the sample solutions within 15 min included peptide fragments digested by ColG. The digestion solutions at 3, 6, 9, 12, and 15 min were mixed in equal amounts and measured by LC-MS. The detected peptides contained a Gly-Xaa-Yaa sequence at the N- or C-terminus. The cleavage sites were mapped on the Col-V sequences (*α*1, *α*2, and *α*3) (Supplementary Figure  1, red triangles; see Supplementary Material available online at http://dx.doi.org/10.1155/2016/4396756). The cleavage sites were distributed in the whole region of the Gly-Xaa-Yaa repeats.

The initial digestion was evaluated by analyzing the short-term digestion. In this digestion experiment, the shortest digestion time was 3 min. The 3 min digestion solution was subjected to LC-MS analysis, and the cleavage site sequences (containing Gly-Xaa-Yaa) were mapped on the same sequences described above (Supplementary Figure  1, blue triangles). The cleavage sites within the 3-min digestion sample were distributed on the N-terminal side of the *α*1 and *α*2 chains. In this experiment, Col-V from human placenta was used. Human placenta contains Col-V in the form of *α*1(V)*α*2(V)*α*3(V) [23]. Thus, the *α*3 chain could be detected. However, the distribution in the *α*3 chain was not clear, because the relative abundance of the detected peptides was much lower than those of the other two chains.

For the *α*1 chain, the modification positions for hydroxyl groups onto proline have been reported [24]. The ColG preference for hydroxyproline (Hyp) at the Yaa position can be calculated by (number of Hyp at the Yaa position within cleavage sites)/(number of cleavage sites) = 29/53 = 54.7%. Meanwhile, the average distribution of Hyp within the Gly-Xaa-Yaa repeats can be calculated by (number of Hyp at the Yaa position)/(number of Gly-Xaa-Yaa repeats) = 97/338 = 28.7%. These results indicated that Hyp residues were preferentially involved at the Yaa position in the cleavage sites.

### 3.4. Long-Term Digestion of Col-V by ColG

**Figure 4:**
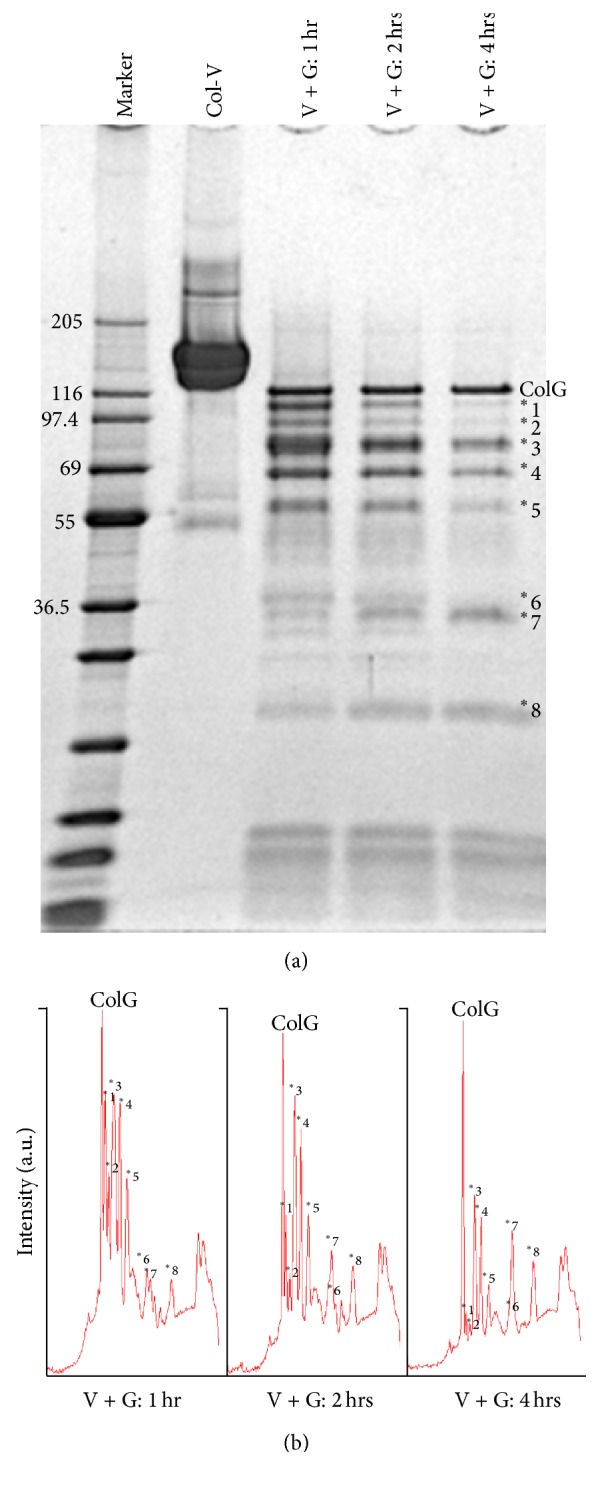
SDS-PAGE analysis of Col-V digestion by ColG over a long time period (~4 h). (a) SDS-PAGE analysis of Col-V digestions. ColG and the main bands are numbered. (b) Band intensity analysis by densitometry.

The short-term digestion of Col-V by ColG revealed an interesting digestion pattern. Therefore, we conducted a long-term digestion experiment to investigate the digestion pattern from 1 to 4 h. The digestion samples were analyzed by SDS-PAGE (Figure 4(a)). On the SDS-PAGE gel, the main bands at >120 kDa disappeared and several bands appeared in the lower molecular weight region. These bands were numbered 1–8. Of these bands, the intensities of bands 1–6 decreased over time (Figure 4(b)). Meanwhile, the lower molecular weight bands (7 and 8) increased in intensity over time. Although the cleavage sites cannot be identified by SDS-PAGE analysis, the appearance of distinct bands indicated the existence of region selectivity for ColG digestion within the Gly-Xaa-Yaa repeats of Col-V. Furthermore, the newly appearing bands also decreased in the long-term digestion, suggesting that there were secondary digestions of the digested fragments.

### 3.5. Tissue Digestion and MS Analysis

#### 3.5.1. Porcine Pancreatic Tissue Digestion

**Figure 5:**
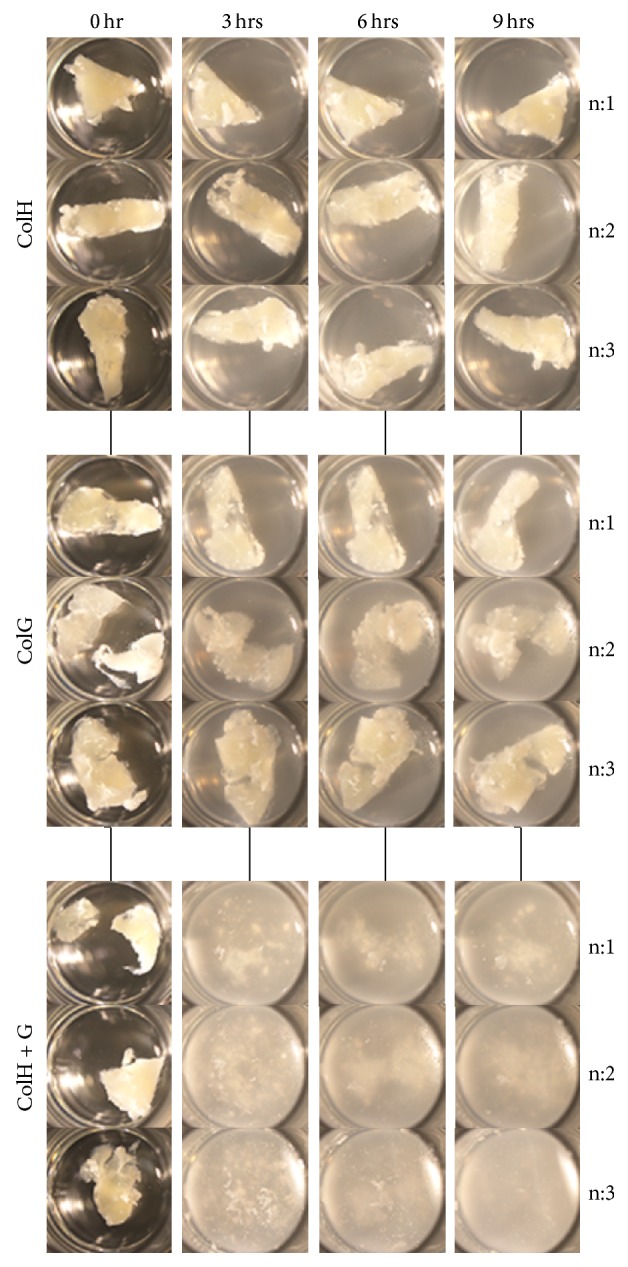
*In vitro* porcine pancreatic tissue digestions by proteases. The digestions were observed at 0, 3, 6, and 9 h. Each digestion was conducted in triplicate.

During pancreatic islet isolation, the exogenous enzymes used are ColH, ColG, and thermolysin [1]. Of these enzymes, thermolysin does not digest collagens under physiological conditions (manuscript in preparation). The porcine pancreatic tissue digestion experiments were carried out in two steps. The first step involved digestion by thermolysin, and the second step involved digestion by ColH, ColG, or ColH + ColG (double digestion). These two-step digestions had significant effects on detecting the collagen peptides in the subsequent mass spectrometric analyses, because the digested peptides by thermolysin could be removed from the solution before the second digestion.  Figure 5 shows the observed pancreatic tissue digestion patterns over time. The tissue samples were not digested by thermolysin alone. To exclude any influence of the proteins digested by thermolysin, the sample solutions were changed after the thermolysin digestion, and the observations were started after addition of the collagenases. Interestingly, the pancreatic tissues were not digested by ColH or ColG alone. Only the solution including both ColH and ColG exhibited tissue degradation. These observations are consistent with our previous findings that both ColG and ColH are necessary for dissociating porcine pancreatic tissues. Although the observations were carried out until 9 h, all three tissue pieces were digested within 3 h.

#### 3.5.2. Col-V Fragments Produced by ColG Digestion of Pancreatic Tissue

The digested samples by ColH and ColG were applied to LC-MS analysis. Database searches focused on identifying collagen peptides were carried out. The identified collagen peptides were picked up from the search results and are summarized in Table 2. Most of the identified peptides were tryptic/semitryptic peptides. Col-V peptides were hardly detected and their sequences were shared by tryptic/semitryptic peptides. It was speculated that Col-V digestion proceeded to unsuitably small fragments in the presence of ColG together with other endogenous proteases for detection of the mass spectrometric analyses.

## 4. Discussion

Our* in vitro* digestion experiments revealed that the Col-V digestion patterns were distinctly different between ColH and ColG.

Col-V digestion by ColG was clearly shown by SDS-PAGE and mass spectrometric analyses. Meanwhile, ColH did not digest Col-V under the same conditions used for ColG. Although this study could not investigate the difference in binding ability toward Col-V between ColH and ColG, it is considered that the difference in domain construction is one of the possible reasons for the different collagenolytic activities of ColH and ColG for Col-V. ColH has only one CBD, while ColG contains tandem CBDs (Figure 1). The CBDs of clostridial collagenases have been investigated in detail. A structural comparison indicated that the two structures were similar (rmsd for C*α* = 1.5 Å), despite the relatively low sequence similarity [11]. The substrate-binding specificity of CBDs is broad for collagen types I–IV [6]. The CBDs of ColH and ColG can bind to triple helical collagens, but not to telopeptides [5, 25]. However, tandem CDBs can bind more efficiently to Col-I than a monomeric CBD [25]. Consequently, the domain construction, especially the CBD arrangement, plays a very important role in the digestion characteristics for Col-V.

In the present study, Col-V digestion by ColG produced distinct digestion characteristics within a short time period (within minutes) and a long time period (within hours). The mass spectrometric analyses revealed that the identified peptide cleavage sites were distributed in the N-terminal region of the Gly-Xaa-Yaa repeat region of Col-V. This finding suggests that the digestion of Col-V is initiated from the N-terminal region. According to the ColG digestion for Col-I [26], Col-I is hydrolyzed near the C-terminal end of the triple helix region, and a second cleavage occurs at the N-terminal side. Although a digestion tendency for the C-terminal side was not found in our results, digestion from the N-terminal region is a common digestion property of ColG for both Col-I and Col-V. The long-term digestion experiment indicated several residual parts of Col-V that appeared as distinct bands at 1 h on SDS-PAGE. These parts were also hydrolyzed by subsequent digestion (~4 h). It is suggested that the Col-V sequence includes some parts that are resistant to collagenolysis.

The tissue digestion experiments clearly showed that both ColH and ColG were needed to degrade the pancreatic tissue structure. For human tissue degradation, it was reported that the ratio of ColG/H influenced the islet isolation outcome [27, 28]. These results indicate that ColG may play more important roles in digestion procedure of pig and human tissues than that of rat tissues. The mass spectrometric analysis for the digested samples identified collagen peptides of Col-IV, Col-V, and collagen type VI. Our previous study revealed that ColH is crucial for rat pancreatic islet isolation, and the most abundantly detected collagen peptides in the mass spectrometric analysis were Col-I and Col-III [17]. These results strongly suggested the presence of marked differences between rat and porcine pancreatic tissue degradations. Although it is difficult to identify the exact reason for the discrepancies, fibril formation could be a possible explanation. Collagen molecules are differently involved in various tissues according to animal species [29]. Col-V is associated with Col-I in the microfibrillar structure [30, 31]. Furthermore, Col-V can be involved in heparin/heparin sulfate modifications [32, 33] and glycosylation [34], as well as the well-known hydroxyl group modifications at proline and lysine residues [35]. Therefore, the differences in circumstances of the Col-I/V fibrillar structures in the rat and porcine pancreatic tissue may influence the pancreatic islet isolation efficiency. Although the similarities of the circumstances of the Col-I/V fibrillar structures among rats, pigs, and humans for pancreatic islet isolation are difficult to compare, ColG appears to be more important in porcine and human islet isolation processes, and Col-V may be one of the key substrates for ColG.

## 5. Conclusions

In this study, we focused on Col-V digestion by clostridial collagenases. Col-V was distinctively digested by ColG. Mass spectrometric analyses revealed that Col-V digestion by ColG proceeded from the N-terminal region. Although both clostridial collagenases, ColH and ColG, include common domains such as the activator domain, peptidase domain, PKD, and CBD, their domain construction is different. The domain construction may play a crucial role for determining the collagen digestion patterns by the clostridial collagenases. The porcine pancreatic tissue digestion experiments demonstrated that both collagenases are needed to degrade the tissue structure. These observations are completely different from those in rat experiments, in which ColG was not crucially needed. Consequently, the present study reveals that ColG as well as ColH plays a crucial role in pancreatic islet isolation in large mammals and that Col-V can be one of the key substrates for ColG.

## Supplementary Material

Supplementary figure 1: Sequences of collagen a1, a2, and a3. The Gly-Xaa-Yaa repeats are denoted in individual block. The red and blue triangles indicate the cleavage sites detected for the sample which is digested 60 min and 3 min, respectively. The hydroxylproline positions in a1(V) are shown in green.

## Figures and Tables

**Table 1:** Peptide numbers detected by ESI-MS.

		# pep	# tryp	# semitryp		# nontryp		% col. pep
				Gxx-G	non-Gxx-G	Gxx-G	non-Gxx-G	
V	*α*1	29, 31	20, 33	1, 2	8, 6	0, 0	0, 0	3, 6
	*α*2	45, 38	30, 25	4, 6	11, 7	0, 0	0, 0	9, 16
	*α*3	17, 17	15, 15	0, 0	2, 2	0, 0	0, 0	0, 0

V + H	*α*1	39, 33	21, 20	9, 6	4, 6	3, 0	0, 0	31, 27
	*α*2	46, 47	26, 30	9, 7	10, 9	1, 1	0, 0	22, 17
	*α*3	21, 18	15, 16	4, 1	2, 1	0, 0	0, 0	19, 6

V + G	*α*1	64, 56	17, 15	17, 16	8, 5	22, 20	0, 0	61, 64
	*α*2	71, 62	22, 19	28, 26	7, 2	14, 15	0, 0	59, 66
	*α*3	34, 24	10, 7	12, 9	1, 1	11, 7	0, 0	68, 67

# pep, # tryp, # semitryp, and # nontryp indicate the number of total peptides, number of tryptic peptides (R/K)↓xxx⋯xxx(R/K)↓xxx⋯, number of semitryptic peptides (R/K)↓xxx⋯ or ⋯xxx(R/K)↓xxx⋯, and number of nontryptic peptides, respectively.

Gxx-G is the preferred substrate sequence for collagenase at position P3-P2-P1-P1′.

Type V collagen was digested by trypsin (V), trypsin + collagenase H (V + H), and trypsin + collagenase G (V + G).

The percentage of collagenolytic peptides was calculated by the following equation: % col. pep = (number of semitryptic Gxx-G + number of nontryptic Gxx-G)/(number of total peptides).

**Table 2:** Collagen peptide identification from tissue digestion samples.

Col type		# tryp + # semitryp	# nontryp	# Gxx-G
Col-V	*α*1	2, 0	0	2
	*α*2	4, 0	0	3

Col-IV	*α*1	1, 2	3	2
	*α*2	1, 1	1	1

Col-VI	*α*1	5, 8	0	0
	*α*2	9, 1	1	0
	*α*3	3, 2	1	0

# tryp, # semitryp, and # nontryp indicate the number of tryptic peptides (R/K)↓xxx⋯xxx(R/K)↓xxx⋯, number of semitryptic peptides (R/K)↓xxx⋯ or ⋯xxx(R/K)↓xxx⋯, and number of nontryptic peptides, respectively.

The total number of detected peptides is shown by # tryp + # semitryp + # nontryp.

# Gxx-G can be shared in # tryp, # semitryp, and # nontryp.

Database searches for porcine collagens were based on the following NCBI accession numbers: *α*1(V): 62461592; *α*2(V): 157427707; *α*1(IV): 927191681; *α*2(IV): 927191683; *α*1(VI): 92020086, 92020094, and 545895133; *α*2(VI): 927203606; *α*3(VI): 92020131 and 927211912.
