# Supplementary material for: Collagen V Is a Potential Substrate for Clostridial Collagenase G in Pancreatic Islet Isolation
Source: J Diabetes Res. 2016 Apr 18;2016:4396756. doi: 10.1155/2016/4396756 (PMC4852369; doi:10.1155/2016/4396756)
Supplement: Supplementary file 1 — Supplementary figure 1: Sequences of collagen a1, a2, and a3. The Gly-Xaa-Yaa repeats are denoted in individual block. The red and blue triangles indicate the cleavage sites detected for the sample which is digested 60 min and 3 min, respectively. The hydroxylproline positions in a1(V) are shown in green. [file 4396756.f1.pdf]

Supplementary figure for

Collagen V is potential substrate for clostridial collagenase G in pancreatic islet isolation

Hiroki Shima, Akiko Inagaki, Takehiro Imura, Youhei Yamagata, Kimiko Watanabe, Kazuhiko Igarashi, Masafumi Goto, Kazutaka Murayama

Col-V α1 sequence

MDVHTRWKARSALRPGAPLLPPLLLLLLWAPPPSRAAQPADLLKVLDFHNLPDGITKTTGFCATRR
SSKGPDVAYRVTKDAQLSAPTKQLYPASAFPEDFSILTTVKAKKGSQAFLVSIYNEQGIQQIGLEL
GRSPVFLYEDHTGKPGPEDYPLFRGINLSDGKWHRIALSVHKKNVTLILDCKKKTTKFLDRSDHPM
IDINGIIVFGTRILDEEVFEGDIQQLLFVSDHRAAYDYCEHYSPDCDTAVPDTPQSQDPNPDEYYT
EGDGEGETYYYEYPYYEDPEDLGKEPTPSKKPVEAAKETTEVPEELTPTPTEAAPMPETSEGAGKE
EDVGIGDYDYVPSEDYYTPSPYDDLTYGEGEENPDQPTDPGAGAEIPTSTADTSNSSNPAPPPGEG
ADDLEGEFTEETIRNLDENYYDPYYDPTSSPSEIGPGMPANQDTIYEGIGGPRGEKGQKGEPAIIE
PGMLIEGPPGPEGPAGLPGPPGTMGPTGQVGDPGERGPPGRPGLPGADGLPGPPGTMLMLPFRFGG
GSDAGSKGPMVSAQESQAQAILQQARLALR

GPAGPMGLTGRPGPVGPPGSGGLKGEPGDVGPQGPRGVQGPPGPAGKPGRRGRAGSDGARGMPGQT

GPKGDRGFDGLAGLPGEKGHRGDPGPSGPPGPPGDDGERGDDGEVGPRGLPGEPGPRGLLGPKGPP

GPPGPPGVTGMDGQPGPKGNVGPQGEPGPPGQQGNPGAQGLPGPQGAIGPPGEKGPLGKPGLPGMP

GADGPPGHPGKEGPPGEKGGQGPPGPQGPIGYPGPRGVKGADGIRGLKGTKGEKGEDGFPGFKGDM

GIKGDRGEIGPPGPRGEDGPEGPKGRGGPNGDPGPLGPPGEKGKLGVPGLPGYPGRQGPKGSIGFP

GFPGANGEKGGRGTPGKPGPRGQRGPTGPRGERGPRGITGKPGPKGNSGGDGPAGPPGERGPNGPQ

GPTGFPGPKGPPGPPGKDGLPGHPGQRGETGFQGKTGPPGPPGVVGPQGPTGETGPMGERGHPGPP

GPPGEQGLPGLAGKEGTKGDPGPAGLPGKDGPPGLRGFPGDRGLPGPVGALGLKGNEGPPGPPGPA

GSPGERGPAGAAGPIGIPGRPGPQGPPGPAGEKGAPGEKGPQGPAGRDGLQGPVGLPGPAGPVGPP

GEDGDKGEIGEPGQKGSKGDKGEQGPPGPTGPQGPIGQPGPSGADGEPGPRGQQGLFGQKGDEGPR

GFPGPPGPVGLQGLPGPPGEKGETGDVGQMGPPGPPGPRGPSGAPGADGPQGPPGGIGNPGAVGEK

GEPGEAGEPGLPGEGGPPGPKGERGEKGESGPSGAAGPPGPKGPPGDDGPKGSPGPVGFPGDPGPP

GEPGPAGQDGPPGDKGDDGEPGQTGSPGPTGEPGPSGPPGKRGPPGPAGPEGRQGEKGAKGEAGLE

GPPGKTGPIGPQGAPGKPGPDGLRGIPGPVGEQGLPGSPGPDGPPGPMGPPGLPGLKGDSGPKGEK

GHPGLIGLIGPPGEQGEKGDRGLPGPQGSSGPKGEQGITGPSGPIGPPGPPGLPGPPGPKGAKGSS

GPTGPKGEAGHPGPPGPPGPPGEV

IQPLPIQASRTRRNIDASQLLDDGNGENYVDYADGMEEIFGSLNSLKLEIEQMKRPLGTQQNPART
CKDLQLCHPDFPDGEYWVDPNQGCSRDSFKVYCNFTAGGSTCVFPDKKSEGARITSWPKENPGSWF
SEFKRGKLLSYVDAEGNPVGVVQMTFLRLLSASAHQNVTYHCYQSVAWQDAATGSYDKALRFLGSN
DEEMSYDNNPYIRALVDGCATKKGYQKTVLEIDTPKVEQVPIVDIMFNDFGEASQKFGFEVGPACF
MG

Suppl. fig. 1

Col-V α2 sequence

MMANWAEARPLLILIVLLGQFVSIKAQEEDEDEGYGEEIACTQNGQMYLNRDIWKPAPCQICVCDN
GAILCDKIECQDVLDCADPVTPPGECCPVCSQTPGGGNTNFGRGRKGQKGEPGLVPVVTGIRGRPG
PAGPPGSQGPRGERGPKGRPGPRGPQGIDGEPGVPGQPGAPGPPGHPSHPGPDGLSRPFSAQMAGL
DEKSGLGSQVGLMP

GSVGPVGPRGPQGLQGQQGGAGPTGPPGEPGDPGPMGPIGSRGPEGPPGKPGEDGEPGRNGNPGEV

GFAGSPGARGFPGAPGLPGLKGHRGHKGLEGPKGEVGAPGSKGEAGPTGPMGAMGPLGPRGMPGER

GRLGPQGAPGQRGAHGMPGKPGPMGPLGIPGSSGFPGNPGMKGEAGPTGARGPEGPQGQRGETGPP

GPVGSPGLPGAIGTDGTPGAKGPTGSPGTSGPPGSAGPPGSPGPQGSTGPQGIRGQPGDPGVPGFK

GEAGPKGEPGPHGIQGPIGPPGEEGKRGPRGDPGTVGPPGPVGERGAPGNRGFPGSDGLPGPKGAQ

GERGPVGSSGPKGSQGDPGRPGEPGLPGARGLTGNPGVQGPEGKLGPLGAPGEDGRPGPPGSIGIR

GQPGSMGLPGPKGSSGDPGKPGEAGNAGVPGQRGAPGKDGEVGPSGPVGPPGLAGERGEQGPPGPT

GFQGLPGPPGPPGEGGKPGDQGVPGDPGAVGPLGPRGERGNPGERGEPGITGLPGEKGMAGGHGPD

GPKGSPGPSGTPGDTGPPGLQGMPGERGIAGTPGPKGDRGGIGEKGAEGTAGNDGARGLPGPLGPP

GPAGPTGEKGEPGPRGLVGPPGSRGNPGSRGENGPTGAVGFAGPQGPDGQPGVKGEPGEPGQKGDA

GSPGPQGLAGSPGPHGPNGVPGLKGGRGTQGPPGATGFPGSAGRVGPPGPAGAPGPAGPLGEPGKE

GPPGLRGDPGSHGRVGDRGPAGPPGGPGDKGDPGEDGQPGPDGPPGPAGTTGQRGIVGMPGQRGER

GMPGLPGPAGTPGKVGPTGATGDKGPPGPVGPPGSNGPVGEPGPEGPAGNDGTPGRDGAVGERGDR

GDPGPAGLPGSQGAPGTPGPVGAPGDAGQRGDPGSRGPIGPPGRAGKRGLPGPQGPRGDKGDHGDR

GDRGQKGHRGFTGLQGLPGPPGPNGEQGSAGIPGPFGPRGPPGPVGPSGKEGNPGPLGPIGPPGVR

GSVGEAGPEGPPGEPGPPGPPGPPGHL

TAALGDIMGHYDESMPDPLPEFTEDQAAPDDKNKTDPGVHATLKSLSSQIETMRSPDGSKKHPART
CDDLKLCHSAKQSGEYWIDPNQGSVEDAIKVYCNMETGETCISANPSSVPRKTWWASKSPDNKPVW
YGLDMNRGSQFAYGDHQSPNTAITQMTFLRLLSKEASQNITYICKNSVGYMDDQAKNLKKAVVLKG
ANDLDIKAEGNIRFRYIVLQDTCSKRNGNVGKTVFEYRTQNVARLPIIDLAPVDVGGTDQEFGVEI
GPVCFV

Suppl. fig. 1

Col-V α3 sequence

MGNRRDLGQPRAGLCLLLAALQLLPGTQADPVDVLKALGVQGGQAGVPEGPGFCPQRTPEGDRAFR
IGQASTLGIPTWELFPEGHFPENFSLLITLRGQPANQSVLLSIYDERGARQLGLALGPALGLLGDP
FRPLPQQVNLTDGRWHRVAVSIDGEMVTLVADCEAQPPVLGHGPRFISIAGLTVLGTQDLGEKTFE
GDIQELLISPDPQAAFQACERYLPDCDNLAPAATVAPQGEPETPRPRRKGKGKGRKKGRGRKGKGR
KKNKEIWTSSPPPDSAENQTSTDIPKTETPAPNLPPTPTPLVVTSTVTTGLNATILEGSLDPDSGT
ELGTLETKAAREDEEGDDSTMGPDFRAAEYPSRTQFQIFPGAGEKGAKGEPAVIEKGQQFEGPPGA
PGPQGVVGPSGPPGPPGFPGDPGPPGPAGLPGIPGIDGIRGPPGTVIMMPFQFAGGSFKGPPVSFQ
QAQAQAVLQQTQLSMK

GPPGPVGLTGRPGPVGLPGHPGLKGEEGAEGPQGPRGLQGPHGPPGRVGKMGRPGADGARGLPGDT

GPKGDRGFDGLPGLPGEKGQRGDFGHVGQPGPPGEDGERGAEGPPGPTGQAGEPGPRGLLGPRGSP

GPTGRPGVTGIDGAPGAKGNVGPPGEPGPPGQQGNHGSQGLPGPQGLIGTPGEKGPPGNPGIPGLP

GSDGPLGHPGHEGPTGEKGAQGPPGSAGPPGYPGPRGVKGTSGNRGLQGEKGEKGEDGFPGFKGDV

GLKGDQGKPGAPGPRGEDGPEGPKGQAGQAGEEGPPGSAGEKGKLGVPGLPGYPGRPGPKGSIGFP

GPLGPIGEKGKSGKTGQPGLEGERGPPGSRGERGQPGATGQPGPKGDVGQDGAPGIPGEKGLPGLQ

GPPGFPGPKGPPGHQGKDGRPGHPGQRGELGFQGQTGPPGPAGVLGPQGKTGEVGPLGERGPPGPP

GPPGEQGLPGLEGREGAKGELGPPGPLGKEGPAGLRGFPGPKGGPGDPGPTGLKGDKGPPGPVGAN

GSPGERGPLGPAGGIGLPGQSGSEGPVGPAGKKGSRGERGPPGPTGKDGIPGPLGPLGPPGAAGPS

GEEGDKGDVGAPGHKGSKGDKGDAGPPGQPGIRGPAGHPGPPGADGAQGRRGPPGLFGQKGDDGVR

GFVGVIGPPGLQGLPGPPGEKGEVGDVGSMGPHGAPGPRGPQGPTGSEGTPGLPGGVGQPGAVGEK

GERGDAGDPGPPGAPGIPGPKGDIGEKGDSGPSGAAGPPGKKGPPGEDGAKGSVGPTGLPGDLGPP

GDPGVSGIDGSPGEKGDPGDVGGPGPPGASGEPGAPGPPGKRGPSGHMGREGREGEKGAKGEPGPD

GPPGRTGPMGARGPPGRVGPEGLRGIPGPVGEPGLLGAPGQMGPPGPLGPSGLPGLKGDTGPKGEK

GHIGLIGLIGPPGEAGEKGDQGLPGVQGPPGPKGDPGPPGPIGSLGHPGPPGVAGPLGQKGSKGSP

GSMGPRGDTGPAGPPGPPGAP

AELHGLRRRRRFVPVPLPVVEGGLEEVLASLTSLSLELEQLRRPPGTAERPGLVCHELHRNHPHLP
DGEYWIDPNQGCARDSFRVFCNFTAGGETCLYPDKKFEIVKLASWSKEKPGGWYSTFRRGKKFSYV
DADGSPVNVVQLNFLKLLSATARQNFTYSCQNAAAWLDEATGDYSHSARFLGTNGEELSFNQTTAT
TVSVPQDGCRLRKGQTKTLFEFSSSRAGFLPLWDVAATDFGQTNQKFGFELGPVCFSS

Suppl. fig. 1

Supplementary figure 1. Sequences of collagen α1, α2, and α3. The Gly-Xaa-Yaa repeats are denoted in individual block. The red and blue triangles indicate the cleavage sites detected for the sample which is digested 60 min and 3min, respectively. The hydroxylproline positions in α1(V) are shown in green.
